# Supplementary material for: Enhanced and stable spin Hall conductivity in a disordered time-reversal and inversion symmetry broken topological insulator thin film
Source: Sci Rep. 2022 Sep 13;12:15379. doi: 10.1038/s41598-022-19756-7 (PMC9470585; doi:10.1038/s41598-022-19756-7)
Supplement: Supplementary file 1 — Supplementary Information. [file 41598_2022_19756_MOESM1_ESM.pdf]

Supplementary Material for "Enhanced and stable spin Hall conductivity in a disordered time-reversal and inversion symmetry broken topological insulator thin film"

Siamak Pooyan and Mir Vahid Hosseini
Department of Physics, Faculty of Science, University of Zanjan, Zanjan 45371-38791, Iran

SOME NOTATIONS

We have introduced $S^{\pm}_{i,j}$ in Eq. (19) of the main text as

$$\begin{aligned}
S^{\pm}_{00} =& V\left(V^2-\left(v_{Fk}^2k^2+(\chi_\pm+\Sigma_{00})^2-(\Delta+\Sigma_{0x})^2\right)\right)-M\left((V-\chi_\pm-\Sigma_{yy})+(\Delta+\Sigma_{0x})^2\right)\\
&+(\chi_\pm+\Sigma_{zz})\left(V^2-\left(-v_{Fk}^2k^2+(\chi_\pm+\Sigma_{xx})^2-(\Delta+\Sigma_{yz})^2\right)\right),\\
S^{\pm}_{xx} =& -V\left(V^2-\left(v_{Fk}^2k^2+(\chi_\pm+\Sigma_{00})^2-(\Delta+\Sigma_{0x})^2\right)\right)+M\left(v_{Fk}^2k^2+(V-\chi_\pm-\Sigma_{yy})\right)\\
&+(\chi_\pm+\Sigma_{zz})\left(V^2-\left(-v_{Fk}^2k^2+(\chi_\pm+\Sigma_{yy})^2-(\Delta+\Sigma_{yz})^2\right)\right),\\
S^{\pm}_{yy} =& V\left(V^2-\left(v_{Fk}^2k^2+(\chi_\pm+\Sigma_{00})^2-(\Delta+\Sigma_{0x})^2\right)\right)-M\left((V-\chi_\pm-\Sigma_{yy})-(\Delta+\Sigma_{0x})^2\right)\\
&+(\chi_\pm+\Sigma_{zz})\left(V^2-\left(-v_{Fk}^2k^2+(\chi_\pm+\Sigma_{xx})^2-(\Delta+\Sigma_{yz})^2\right)\right),\\
S^{\pm}_{zz} =& -V\left(V^2-\left(v_{Fk}^2k^2+(\chi_\pm+\Sigma_{00})^2-(\Delta+\Sigma_{0x})^2\right)\right)-M\left(v_{Fk}^2k^2-(V-\chi_\pm-\Sigma_{yy})\right)\\
&+(\chi_\pm+\Sigma_{zz})\left(V^2-\left(-v_{Fk}^2k^2+(\chi_\pm+\Sigma_{yy})^2-(\Delta+\Sigma_{yz})^2\right)\right),\\
S^{\pm}_{0x} =& -(\Delta+\Sigma_{0x})\left(v_{Fk}^2k^2+V^2-M(-V+\chi_\pm+\Sigma_{00})-(\chi_\pm+\Sigma_{xx})^2+(\Delta+\Sigma_{0x})^2\right),\\
S^{\pm}_{yz} =& -(\Delta+\Sigma_{yz})\left(v_{Fk}^2k^2+V^2+M(-V+\chi_\pm+\Sigma_{yy})-(\chi_\pm+\Sigma_{zz})^2+(\Delta+\Sigma_{yz})^2\right).
\end{aligned}$$
